# The Role of Ga-68 PSMA PET/CT Scan on Differentiating of Oligometastatic and High Risk Prostate Cancer

**DOI:** 10.4274/mirt.galenos.2020.89421

**Published:** 2020-10-19

**Authors:** Mehmet Erdoğan, Emine Elif Özkan, Sefa Alperen Öztürk, Mustafa Yıldız, Sevim Süreyya Şengül

**Affiliations:** 1 Süleyman Demirel University Faculty of Medicine, Department of Nuclear Medicine, Isparta, Turkey; 2 Süleyman Demirel University Faculty of Medicine, Department of Radiation Oncology, Isparta, Turkey; 3 Süleyman Demirel University Faculty of Medicine, Department of Urology, Isparta, Turkey

**Keywords:** Ga-68 PSMA PET/CT, prostate cancer, oligometastasis, SUVmax

## Abstract

**Objectives:**

In this study, we aimed to investigate whether Ga-68 prostate-specific membrane antigen positron emission tomography/computed tomography (PSMA PET/CT) scanning is adequate to predict intermediate risk, high risk, or oligometastatic prostate cancer (PCa) as an initial staging modality.

**Methods:**

The Ga-68 PSMA PET/CT scan images of 50 PCa patients pathologically proven by transrectal ultrasound guided biopsy were evaluated retrospectively. The association of standard uptake value maximum (SUV_max_) value of the area with the highest PSMA expression within the primary tumor with the risk groups and metastatic burden is investigated.

**Results:**

The SUV_max_ value was 6.18 in oligometastatic patients where it was measured as 10.93 in patients with higher metastatic burden (p=0.037). The cut-off SUV_max_ value for multiple metastases was 7.96 (p=0.047). According to the regression model, SUV_max_ value has a positive influence [odds ratio (OR)=1.42], which was statistically significant (p=0.038). SUV_max_ values for intermediate and high risk patients were 6.91 and 11.44, respectively (p=0.014). The cut-off SUV_max_ value for the high risk group was 10.55 (p=0.006). In the regression model, SUV_max_ value has a positive influence (OR=1.198), which was statistically significant (p=0.021).

**Conclusion:**

In this paper, we demonstrated the association between SUV_max_ value of primary tumor and Gleason score. Our results also allowed us to suggest that primary tumor SUV_max_ is a sufficiently accurate predictor of D’Amico risk groups in newly diagnosed PCa cases. Additionally, Ga-68 PSMA PET/CT turns out to be a useful tool in determining oligometastatic PCa, which requires a different treatment approach.

## Abstract (tr)

**Amaç:**

Çalışmamızda, Ga-68 prostat spesifik membran antijen pozitron emisyon-bilgisayarlı tomografi (PSMA PET/BT) taramanın maksimum standardize uptake değerini (SUV_maks_), başlangıç evrelemede orta ve yüksek riskli prostat kanserini (PCa) tahmin edebilir mi? Oligometastatik PCa’yi tahmin edebilir mi? sorularına cevap aradık.

**Yöntem:**

TRUS-Bx temelinde PCa tanısı almış ve Ga-68 PSMA PET/BT tarama yapılmış 50 hastanın görüntüleri retrospektif olarak incelendi. Primer tümörde PSMA ekspresyonunun en yüksek olduğu alanın SUV_maks_ değeri ile risk gruplarının ve metastaz durumunun korelasyonu yapıldı.

**Bulgular:**

Oligometastatik hastalarda SUV_maks_ değeri 6,18 iken, multipl metastazı olan olgularda 10,93 olarak bulundu (p=0,037). Multipl metastaz için cut-off SUV_maks_ değeri 7,96 olarak bulundu (p=0,047). Regresyon modelinde, SUV_maks_’nın katkısı pozitif yönlü [göreceli olasılıklar oranı (OR)=1,42] ve anlamlı bulundu (p=0,038). Orta risk grubunda SUV_maks_ değeri 6,91 iken, yüksek risk grubunda 11,44 olarak bulundu (p=0,014). Yüksek risk grubu için cut-off SUV_maks_ değeri 10,55 olarak bulundu (p=0,006). Regresyon modelinde, SUV_maks_’nın katkısı pozitif yönlü (OR=1,198) ve anlamlı bulundu (p=0,021).

**Sonuç:**

Bu çalışma ile yeni tanı almış PCa’da primer tm’nin SUV_maks_ değerinin, Gleason skoru ile korele olduğunu gösterdik. SUV_maks_’nin, D’Amico risk sınıflamasına göre orta ve yüksek riskli PCa’sı yüksek doğrulukla tahmin edebileceğini düşünüyoruz. SUV_maks_’nin tedavi yaklaşımı açısından önem arz eden oligometastatik PCa’sı yüksek doğrulukla tahmin edebileceğini düşünüyoruz.

## Introduction

Prostate cancer (PCa) is the second most frequent cancer in men and the cause of 5.2% of all cancer-related deaths (1). Prostate-specific antigen (PSA), digital rectal examination, Gleason score (GS), and specific imaging modalities are the most widely used parameters for initial clinical staging. These specific imaging tools are transrectal ultrasound (TRUS), multiparametric magnetic resonance imaging, thoracoabdominal computed tomography (CT), and bone scan (2). The goal of clinical staging in PCa is to determine the burden of disease and predict the prognosis via pretreatment clinical parameters to direct the patient for the most appropriate treatment plan. Procedures to be chosen for staging are specified according to risk stratification. The most widely used risk grouping for PCa is the one defined by D’Amico (3).

Ga-68 prostate-specific membrane antigen positron emission/CT (PSMA PET) scanning in PCa is found to have a higher sensitivity and specificity in distant lymph node metastasis and bone metastasis according to conventional imaging modalities (4). PSMA is a type II transmembrane glycoprotein consisting of 750 amino acids (5). It shows little or no expression in normal prostate cells, whereas it is significantly expressed in prostate carcinoma or metastasis (6). Besides, although it does not enter the circulation, PSMA is an ideal molecular target for nuclear medicine procedures with Ga-68 PSMA PET/CT (7).

As determining oligometastatic patients became crucial in terms of individualizing treatment strategy, PSMA PET/CT became increasingly used as initial staging modality. Hellman and Weichselbaum (8) first suggested the definition of the term “oligometastasis-oligometastatic” in 1995 that means “low burden metastatic patients whose prognostic features are between localized and metastatic disease”. However, a consensus was not constituted on the final definition of oligometastatic disease. Some authors use only the number of metastases, whereas others consider both the number and localization (9).

The Chemo-Hormonal Therapy Versus Androgen Ablation Randomized Trial for Extensive Disease (CHAARTED) study suggested the widely accepted definition in the literature. Patients were stratified as high-volume disease in the presence of visceral metastases or four bone lesions with at least one beyond the vertebral bodies and pelvis and low-volume disease if out of high-volume definition (10). Radical treatment strategies such as surgery or stereotactic radiotherapy may be appropriate alternatives for a limited number of metastatic lesions that are so-called oligometastatic (11). Several studies reported increased overall survival with radical treatment approaches in oligometastatic PCa patients. Therefore, differentiating oligometastatic from multimetastatic disease during initial staging is important.

In this study, we aimed to investigate whether Ga-68 PSMA PET/CT scanning is adequate to predict the risk group or metastatic burden in PCa as an initial staging modality.

## Materials and Methods

### Patients

Images of 50 PCa patients who were diagnosed with 12-24 core TRUS-biopsy were retrospectively investigated. The patients had suspicious metastatic lesions in the bone scan or other conventional imaging techniques and underwent Ga-68 PSMA PET/CT for initial staging. Patients who underwent transurethral resection or radical prostatectomy were excluded.

The Scientific Research Ethics Committee of Medical Faculty of the Süleyman Demirel University (desicion no: 177, 21.05.2019) approved the study. All procedures were performed in terms of the ethical standards of the institutional research committee in alliance with the 1964 Helsinki Declaration and its later amendments. Informed consent was waived owing to the retrospective nature of the study. Pretreatment PSA values of patients were obtained from their electronical charts, and time between PSA test and Ga-68 PSMA PET/CT was maximum of 45 days. Biopsy specimens were reported according to the GS system and Gleason grade system suggested by The International Society of Urological Pathology in 2014 (12): Grade group 1 (GS ≤6), Grade group 2 (GS 3+4=7), grade group 3 (GS4+3=7), Grade group 4 (GS 4+4=8.3+5=8.5+3=8), and grade group 5 (GS 9-10). Consequently, patients were stratified according to D’Amico risk grouping, which classified Gleason grade groups 2 and 3 as intermediate risk group and Gleason grade groups 4 and 5 as high risk group (3). Gleason grade group 1, which is the low risk group, was excluded in these two groups. Patients were then divided into three groups: non-metastatic, oligometastatic, and multimetastatic. Three or less metastatic lesions none or only one of them out of pelvis or vertebra was accepted as oligometastasis as in CHAARTED trial (10). Four or more bone metastasis and lymph node metastasis were included in the multiple metastatic group. None of the patients had visceral metastasis.

### Image Acquisition and Analysis

Images were gathered via Philips Time of Flight PET/CT camera. PET/CT images were obtained 60 min after intravenous injection of 111-185 MBq (3-5 mCi) Ga-68 PSMA ligand. A low-dose CT scan was performed before PSMA PET/CT for attenuation correction and anatomic localization purposes and, consequently, a 3-min caudocranial PET emission scanning in the supine position. CT data were used for attenuation correction, and image reconstruction was done via the standard recursive algorithm. Transaxial, coronal, and sagittal plans were reformed. Maximum intensity projection images were also obtained. Two experienced nuclear medicine specialists evaluated the PET/CT fusion images. (The interrater agreement was high, and ICC=0.926). The highest standard uptake value maximum (SUV_max_) value calculated from the whole prostate tissue is accepted as the highest region of PSMA expression, and it was recorded. Whole body scan was reviewed, especially for bony structure and abdominopelvic lymph nodes. The number and localization of PSMA-expressing bony structures and lymph nodes were also recorded.

### Statistical Analysis

The statistical analysis was performed using SPSS 20.0 (IBM Inc., Chicago, IL, USA) software. Descriptive statistics were presented as frequency (percent ratio) for categorical variables and median; interquartile range for numeric variables. Normal distribution evaluation of PSA and SUV_max_ values were analyzed by Kolmogorov-Smirnov test, and both variables revealed non-parametric results. Therefore, the comparisons were performed by Mann-Whitney U and Kruskal-Wallis tests. Post-hoc analysis of significant results is shown in the tables by superscript letters. Receiver operating characteristic (ROC) analysis was performed for SUV_max_ values to calculate the diagnostic ratios. All tests are presented as two sided with 95% confidence intervals and relevant p values (p<0.05). The association between SUV_max_ values of primary tumor and these two risk groups is statistically analyzed, and logistic regression analysis was also performed. Subgroups of intermediate risk (Gleason grades 2 and 3) were additionally analyzed with each SUV_max_ values of the primary tumor in terms of association. The power analysis was not performed because of the small study sample size. In the SUV_max_ comparisons according to grade categories, power and partial eta square values were 0.853 and 0.200, respectively. Therefore, it appeared that the sample size was sufficient, and 20% of the variance according to categories was clarified.

## Results

Fifty patients were enrolled to our study. The median age was 67.50 (12.25) years. When categorized, the distribution of age ranges was <55 (4%), 55-65 (40%), 65-75 (34%), and >75 (22%) years. More than half (58%) of patients were metastatic. Lymph node metastasis was found in 34% of patients, bone metastasis 40%, and both 16%. Of patients, 1 (2%), 9 (18%), 11 (22%), 15 (30%), and 14 (28%) were reported as grade groups 1, 2, 3, 4, and 5 respectively. No statistically significant difference was found between the Gleason grade groups. According to D’Amico risk classification, 20 (40%) patients were intermediate risk, whereas 29 (58%) were in the high risk group. Seven (14%) patients were found to be oligometastatic, and multiple bone and lymph node metastases were seen in 13 (26%) and 9 (18%) patients, respectively. Patients were also divided into nonmetastatic (42%), oligometastatic (14%), and multimetastatic (44%) groups.

Demographic features of patients are shown in Table 1.

SUV_max_ values in patients with positive biopsy ratio of >50% were higher, but the difference was nonsignificant. When three PSA groups (<10, 10-20, and >20 ng/mL) were analyzed, SUV_max_ values increased with higher PSA values, and this difference was statistically significant (p=0.011). No statistically significant difference was found between the SUV_max_ values of metastatic and non-metastatic patients. The median SUV_max_ value was 10.93 (14.94) and 6.18 (2.49) in multiple metastatic and oligometastatic groups, respectively, which is statistically significant (p=0.037). In intermediate and high risk patients, the SUV_max_ values were 6.91 (3.54) and 11.44 (14.83), respectively, which was also statistically significant (p=0.014). The difference in the SUV_max_ values between grade groups 2 and 3 was not statistically significant (p=0.056). SUV_max_ values in patients with vesicula seminalis invasion were significantly higher (p=0.001; Table 1).

**Figure 1:**
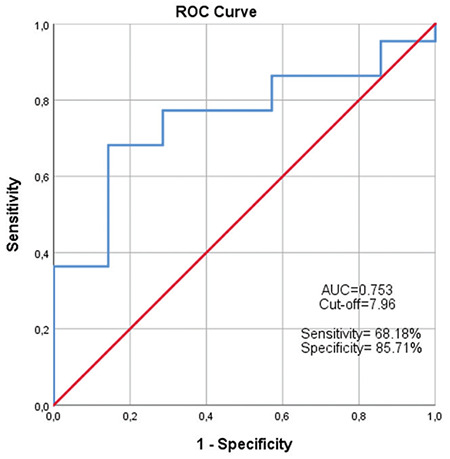
Receiver operating characteristic curve of standard uptake value on oligometastasis and multimetastasis ROC: Receiver operating characteristic, AUC: Area under the curve

Although SUV_max_ values in oligometastatic and multiple metastatic cases were significantly different, ROC analysis was performed. Area under the curve was significant 0.753 (p=0.047), and the cut-off value for SUV_max_ was 7.96 (Figure 1). The sensitivity and specificity of this cut-off value for predicting multiple metastases were 68.18% and 85.71%, respectively. The positive predictive value (PPV) was high as 93.75%, whereas the negative predictive value (NPV) was only 46.15% (Table 2).

**Figure 2:**
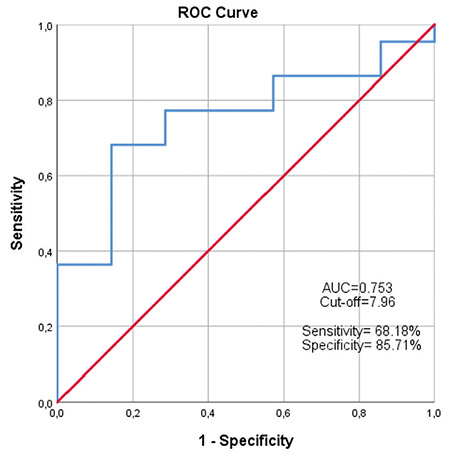
Receiver operating characteristic curve of standard uptake value on intermediate and high risk groups ROC: Receiver operating characteristic, AUC: Area under the curve

ROC analysis was also performed for the risk groups. Area under the curve was statistically significant at 0.727 (p=0.006). The cut-off value for SUV_max_ was 10.55 (Figure 2). The specificity and PPV of this cut-off value for predicting high risk group was 90.00%. The sensitivity and NPV were similar and found to be 62.07% (Table 3).

PSA values were not significantly different between age groups. In contrast, it was significantly higher in patients with positive biopsy ratio of >50% (p=0.002). In patients with lymph node metastasis, the PSA value was significantly higher than patients in the non-metastatic group (p<0.001). The difference was also statistically significant between the oligometastatic and multiple metastatic group (p=0.015), which is higher in multiple metastatic patients. No significant difference was found between the PSA values of D’Amico risk groups. However, it was significantly higher in patients with vesicula seminalis invasion (p=0.006; Table 1).

Univariate logistic regression model was performed between oligometastatic and multiple metastatic patients. PSA and SUV_max_ were the variables to be specified as factors. Oligometastasis is investigated as a reference category. Goodness of fit results for this model were found to be significant and acceptable (-2LL=21.406; Hosmer & Lemeshow X^2^=7.207 (p=0.514). The explanatory ratio of these factors to the multiple metastasis category was sufficiently high (Nagelkerke R^2^=0.459). The contribution of SUVmax to the model was positive [odds ratio (OR=1.42) and significant (p=0.038; Table 4).

Univariate logistic regression model was also used between D’Amico risk groups. PSA, SUV_max_, and age were the variables to be specified as factors. The intermediate risk group was the reference category. Goodness of fit results for this model were found significant (-2LL=51.698; Hosmer & Lemeshow X^2^=4.491 (p=0.810), and the explanatory ratio was R^2^=0.347. Contribution of SUV_max_ to the model was positive (OR=1.198) and significant (p=0.021; Table 4).

## Discussion

**Figure 3:**
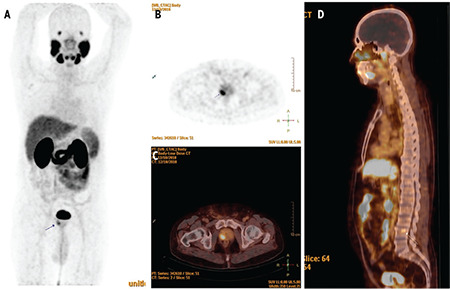
The prostate-specific membrane antigen positron emission (Ga-68 PSMA PET) image of 67 years old prostate carcinoma patient (A), transraxial PET/computed tomography (CT) image of Ga-68 PSMA expressing primary tumor in right lobe apex (B), transraxial PET/CT fusion image (C), sagittal plan PET/CT image of metastatic lesion in the lumbar 4^th^ spine expressing Ga-68 PSMA (D) PSMA: Prostate-specific membrane antigen, PET: Positron emission tomography, CT: Computed tomography

Low burden metastatic PCa is considered to have a different behavioral pattern compared with high burden multiple metastatic counterparts. Although some reports have shown an outcome improvement with radical strategies, optimal treatment approach is still a matter of debate. Moreover, it is a controversy whether it is appropriate to perform aggressive modalities such as surgery or high-dose radiotherapy for both metastasis and primary lesions (13). Considering these discussions to constitute the most appropriate individual approach for low metastatic burden disease, we searched for an answer whether Ga-68 PSMA PET/CT scanning is adequate to predict intermediate risk, high risk, or oligometastatic PCa as an initial staging modality in this study (Figure 3).

According to our results, median SUV_max_ values in oligometastatic and multiple metastatic patients were 6.18 and 10.93, respectively, and this was statistically significant (p=0.037). Further ROC analysis revealed a cut-off value of 7.96 for SUV_max_, and values higher than this predicted high burden disease with a sensitivity, specificity, PPV, and NPV of 68.18%, 85.71%, 93.75%, and 46.15%, respectively. To our knowledge, this is the first study to investigate the SUV_max_ value in Ga-68 PSMA PET/CT in staging and determining the disease burden (oligometastatic or multimetastatic) for PCa patients.

Currently, D’Amico risk group is still the main clinical feature directing treatment decision. Therefore, some studies investigated the association of SUV_max_ with these risk groups. An example of these studies is published by Uprimny et al. (14). The authors found mean SUV_max_ values in intermediate and high risk groups as 8.25 and 20.5, respectively, in their 82-patient sample, which was statistically significant (p<0.001). In another study by Sachpekidis et al. (15) in 24 patients, SUV_max_ values of low and intermediate risk groups were significantly lower compared with that of high risk group in concordance with our study. Likewise, Demirci et al. (16) also found that high risk group had significantly higher SUV_max_ value (p<0.001).

In our study, median SUV_max_ values of intermediate and high risk groups were 6.91 and 11.44, respectively, which were statistically significant (p=0.014), in accordance with previous reports. Related SUV_max_ cut-off value calculated via ROC analysis was 10.55. The specificity and PPV were 90%, and sensitivity and NPV were both 62.07% for the predicting risk group. Demirci et al. (16) found SUV_max_ cut-off value as 9.1 for high risk group, which is close to ours. The difference between SUV_max_ values of Gleason grade groups 2 and 3 was not statistically significant (medians 7.45 and 6.38, respectively) in the same line with the results of several previous studies (14,15). However, Demirci et al. (16) reported significant difference between SUV_max_ values of grades 2 and 3 subgroups. This discordance may be attributed involvement of post radical prostatectomy specimens rather than biopsy when the contradiction between GS reported with biopsy and prostatectomy is considered (17).

As a secondary aim, SUV_max_ values were investigated according to three PSA subgroups. SUV_max_ values increased with increasing PSA, and the difference was found to be statistically significant (p<0.001). Reports addressing the same issue (14,15) also found that primary tumor SUV_max_ value was significantly higher in patients with PSA ≥10 ng/mL.

### Study Limitations

Our study has limitations specific to the retrospective design. Although this study was conducted in a small environment in a restricted time, a small sample size fulfilling the inclusion criteria was enrolled. As a last limitation, the GSs were not verified with radical prostatectomy specimens, although it was not available for all patients.

## Conclusion

Eventually, this study is rewardable in particularly two main aspects. First, we have shown that primary tumor SUV_max_ value in initial Ga-68 PSMA PET/CT would predict the D’Amico risk group with high accuracy, which is to date the main directory of treatment algorithm. Second, to our concern, our study is the first to prove the high accuracy of SUV_max_ and determine a cut-off value for predicting oligometastatic and multimetastatic PCa. In the era of radical approaches for oligometastatic disease, this is crucial for individualizing treatment approach. Further studies with large samples addressing the prognostic value of SUV_max_ on differentiating oligometastatic and multimetastatic PCa, and its prognostic roles are warranted.

## Figures and Tables

**Table 1:**
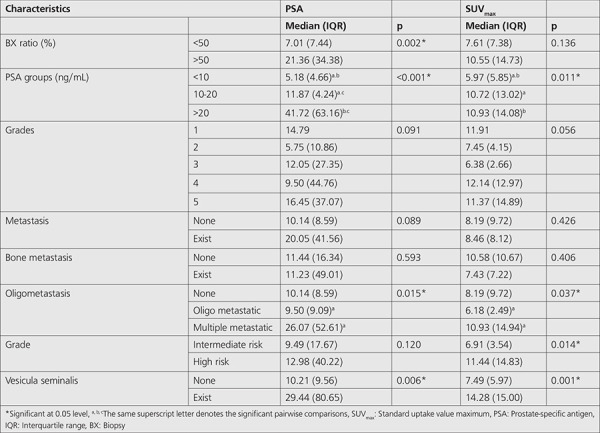
SUV_max_ and PSA according to some characteristics of patients

**Table 2:**
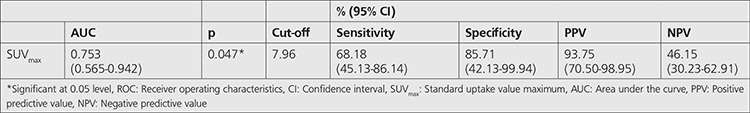
ROC analysis results and diagnostic ratios between oligometastatic and multiple metastatic groups

**Table 3:**
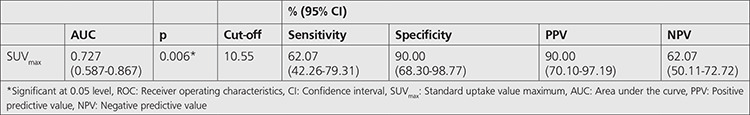
ROC analysis results and diagnostic ratios between intermediate and high risk groups

**Table 4:**
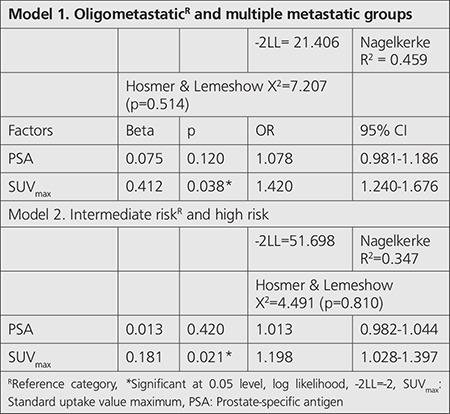
Predictive model of factors affecting metastasis and risk groups
